# Portal Vein Thrombosis (PVT) Secondary to Protein C Deficiency in a Young Male

**DOI:** 10.7759/cureus.49688

**Published:** 2023-11-29

**Authors:** Farhan Zeb, Hidayat Ullah, Arya Harikrishna, Wissam Khalaf, Noman Salih, Amama Waheed, Rooh Ul Amin

**Affiliations:** 1 Medicine, Hayatabad Medical Complex Peshawar, Peshawar, PAK; 2 Medical C Unit, Hayatabad Medical Complex Peshawar, Peshawar, PAK; 3 Medicine, European University Cyprus, Nicosia, CYP; 4 Psychiatry, Psychology and Neuroscience, King's College London, London, GBR; 5 Medicine, Medical University of Lublin, Lublin, POL; 6 Internal Medicine, Hayatabad Medical Complex Peshawar, Peshawar, PAK; 7 Internal Medicine, Khyber Girls Medical College Peshawar, Peshawar, PAK

**Keywords:** triphasic ct scan, doppler ultrasound, anticoagulant therapy, hypercoagulable disorders, portal vein thrombosis

## Abstract

A disorder known as portal venous thrombosis (PVT) is characterized by a partial or total obstruction of the portal vein. Although PVT is somewhat uncommon, liver illness is frequently linked to it. Cirrhosis, carcinoma of the liver, myeloproliferative neoplasms, other malignancies, the use of oral contraceptives, intestinal infections, and genetic hypercoagulable illnesses are typical risk factors. In this case report, we discuss the case of a young male patient who had PVT as a result of a protein C deficit. The patient first had abdominal distention due to ascites, and the diagnosis of portal vein thrombosis was later confirmed by a triphasic computed tomography (CT) scan and Doppler ultrasonography. Anticoagulant medications were successfully administered to treat the patient. The importance of identifying PVT in patients with hypercoagulable diseases and the efficacy of anticoagulant therapy in such circumstances are both highlighted by this case.

## Introduction

A thrombus present inside the portal vein's lumen causes portal vein thrombosis (PVT), which is the partial or total restriction of blood flow in the vein [1]. When Balfour and Stewart originally described the first instance of PVT in 1868, the patient had symptoms such as splenomegaly, ascites, and variceal dilatation [2]. With mean age-standardized prevalence and incidence rates of 3.7 and 0.7 per 100,000 people, respectively, PVT is rather uncommon in the general population [3]. These rates, which range from 4.4% to 15% in people with cirrhosis, considerably increase and represent around 5%-10% of all instances of portal hypertension [4]. Local variables account for 10%-50% of instances with prothrombotic disorders, which appear in 22%-70% of those without cirrhosis [3-5] and frequently involve numerous contributory factors [6].

PVT can present variably in individuals depending on whether it has an acute or chronic start, as well as how much collateral circulation develops. Abdominal discomfort, diarrhea, bleeding from the rectal region, abdominal distention, vomiting, nausea, anorexia, fever, lactic acidosis, septicemia, and splenomegaly are common symptoms of acute PVT, which often results in intestinal congestion and ischemia. The symptoms of chronic PVT, however, might range from complete asymptomaticism to the development of ascites, varices, splenomegaly, and pancytopenia [2].

PVT can be classified into four primary categories: (1) thrombosis restricted to the portal vein above the point where the splenic vein alongside the superior mesenteric vein (SMV) converge; (2) a spreading thrombus into the SMV with open mesenteric veins; (3) diffuse splanchnic venous thrombosis, typically involving large collaterals; and (4) extensive splanchnic venous thrombosis, but only with fine collaterals. Although it may potentially have etiological and prognostic importance, this anatomical categorization is presently most often employed to assess operability. In contrast to individuals with isolated PVT, those with thrombus interfering with the mesenteric vasculature are more likely to experience intestinal infarction and have a decreased risk of variceal bleeding. Patients should be checked for any underlying thrombophilic disorders in every occurrence of PVT. Mutations in the prothrombin and factor V genes, in addition to deficits in naturally occurring anticoagulant proteins such as antithrombin, S, or C, are examples of hereditary thrombophilias that predispose people to PVT. A study by Fisher et al. [7] examined a total of 29 adult patients with hypertension at the portal vein brought on by PVT and discovered that 62% of them had a deficiency in at least one of these naturally occurring anticoagulant proteins. Of these, 28% had deficiencies in both C and S proteins, 31% in both C and antithrombin, 24% in protein S and antithrombin, and 21% in combination.

## Case presentation

A 17-year-old male who was previously healthy and had no known comorbidities visited our Outpatient Department. His main complaints were stomach pain and developing abdominal distention, both of which had been present for the previous four months. He developed his symptoms gradually and persistently and without any improvement with medication. He also claimed to have nausea, but there was no vomiting. It's important to note that neither the patient nor her family had any personal or family history of venous thromboembolism. There had been no abdominal trauma in the patient. A review of the systems revealed constipation.

Upon examination, the patient showed no encephalopathy symptoms and was attentive and well-oriented. His vital signs were also normal. There were no notable discoveries during the general physical examination. An enlarged and bloated abdomen with shifting dullness to percussion was discovered during the gastrointestinal examination. Additionally, palpation revealed splenomegaly. The heart, lungs, throat, and skin were examined, but no substantial abnormalities were found.

Laboratory tests revealed that the patient's hemoglobin level was normal, but his erythrocyte sedimentation rate was slightly increased. Initial laboratory examinations, as shown in Table 1, did not find any significant abnormalities. The serum chemistry results and urine tests were unremarkable. The levels of lipase and amylase were also normal, ruling out any possibility of pancreatitis.

**Table 1:** Baseline Investigations Abbreviations: Hb; hemoglobin, WBC; white blood cells, MCV; mean cell volume, K; potassium, Na; sodium, LDL; low density lipoprotein, TAG; triacyl glycerol, Creat; creatinine, BUN; blood urea nitrogen

Test	Reference value	Patients value
Platelets (/µL)	150000-450000	145500
Hb (g/dL)	11.5-17.5	12.9
MCV (fL)	80-100	78.5
Wbcs (/µL)	4000-11000	11200
Lipase (IU/L)	0-160	35
Amylase (IU/L)	40-140	56
K (mmol/L)	3.6-5.0	4.28
Na (mmol/L)	135-150	143.9
TAG (mg/dL)	<145	98
LDL cholesterol (mg/dL)	<90	56
Creat (mg/dL)	0.4-0.9	0.48
Bun (mg/dL)	18-45	35

The international normalized ratio (INR) was inside the normal range. Results from viral profiles and tumor markers such as CA-19-9, ACE, along alpha-fetoprotein (AFP) were negative. The coagulation profile along with liver function tests are shown in Table 2.

**Table 2:** Liver Function Tests and Coagulation Profile Abbreviations: aPTT; activated partial thromboplastin time, ALP; alkaline phosphatase, ALT; alanine aminotransferase, AST; aspartate aminotransferase; INR; international normalized ratio, PT; prothrombin time

Test	Reference levels	Patient levels
Ast (U/L)	8-33	48
Alt (U/L)	10-50	65
ALP (U/L)	<390	293
Total bilirubin (mg/dL)	0.1-1	0.9
INR	1	0.98
PT (sec)	<13	15.8
aPTT (sec)	<28	27
Protein C activity (%)	70-140	50
Protein S activity (%)	65-140	95
Antithrombin III (%)	74-126	75
Anti-cardiolipin antibodies (IU/mL)	<10	1
Anti B-2 Glycoprotein antibodies (IU/mL)	<5	1

**Figure 1:**
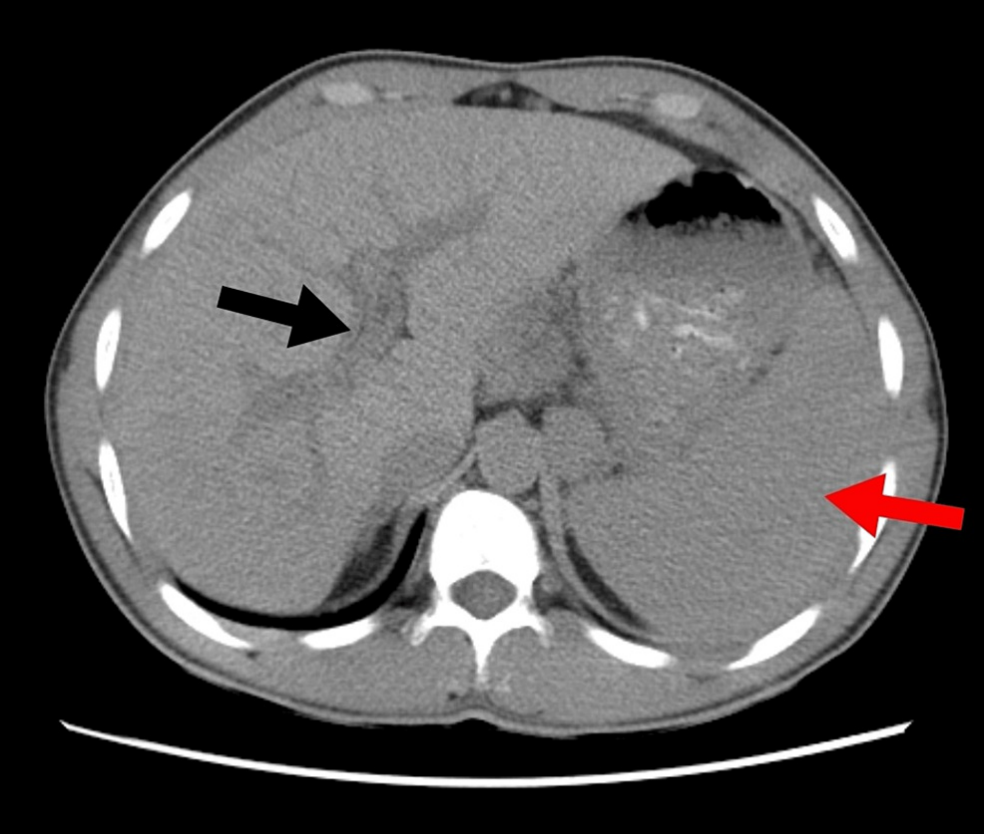
Black Arrow Showing Dilated Portal Vein With Red Arrow Showing Splenomegaly

A subsequent abdominal and pelvic ultrasound revealed substantial ascites and splenomegaly, although the liver's texture looked normal. Owing to this, portal vein thrombosis was suspected. To rule out probable ascites causes, a thorough workup was performed that included evaluations of the patient's liver, kidney, and heart health and dietary factors. Serum-ascites albumin gradient testing and normal ascitic fluid evaluation revealed a high SAAG and low protein level, suggesting portal hypertension. Doppler tests verified that portal vein thrombosis existed. To exclude malignancy and look for any potential external compressive masses affecting the portal vein, a triphasic computed tomography (CT) scan of the abdomen and pelvis was also conducted. Figure 1 from the data illustrates a dilated portal vein, esophageal varices, splenomegaly, dilated splenic vein, inferior vena cava, as well as many varices, all of which are signs of portal hypertension.

There were attempts to pinpoint the origin of the clot. Cardiolipin and antiphospholipid lipid antibodies produced negative findings. The results of a thrombophilia workup, which did not include testing for the JAK2V617F mutation, revealed normal blood levels of homocysteine. Mildly raised levels of C-reactive protein were found, along with indications of decreased protein C and normal protein S antigen activity. The levels of antithrombin III levels were almost normal, and the Factor V Leiden mutation was unremarkable. Therefore, insufficient protein C came out to be the primary cause of PVT. The patient was prescribed a course of therapy that includes weekly doses of 1 mg/kg of enoxaparin, a low molecular weight heparin, and then 2.5 mg of warfarin every day for a long period to target an INR range of 2-3. The patient was then booked for a follow-up appointment, where he showed appreciable changes in his symptoms.

## Discussion

The extensive use of ultrasound-Doppler technology has led to an increase in the frequency of PVT diagnoses in recent years [8]. The lifetime risk of acquiring PVT in people, in general, is believed to be about 1% in situations where fibrosis is not a factor. The recognized etiologies of PVT may be roughly divided into two categories: thrombophilic diseases and thromboses brought on by local causes. An essential component of the coagulation process, protein C functions as an anticoagulant enzyme, inactivating coagulation components V and VIa and activating fibrinolysis [9]. An autosomal dominant characteristic that is often inherited, protein C deficiency is a risk factor for thrombosis of the venous system as occurred in our case.

According to plasma levels alone, it is predicted that one in 200-500 people in the population as a whole have protein C deficiency. These figures, however, may not be accurate as many afflicted people live symptom-free lives. However, between 2% and 5% of individuals with VTE had protein C deficiency. Only one in 50,000-700,000 live births experience more severe instances of the condition, such as homozygous and complex heterozygous protein C deficiency, where the frequency is exceedingly rare.

According to research, genetic thrombophilia may be linked to uncommon thrombosis in young people, including Budd-Chiari syndrome, cerebral venous thrombosis, mesenteric vein thrombosis, and suprahepatic vein thrombosis. The low levels of protein C in our patient cannot be attributed to liver function impairment, which is frequently a result of PVT, as all the tests for liver function were normal. Regarding protein C deficit in PVT patients, studies from diverse areas have revealed varying findings, with some highlighting a high frequency and others focusing on protein S deficiency or both [10,11]. Investigation of possible inherited anticoagulant protein deficiency, which may be validated by a thorough review of the patient's family history, is crucial in PVT instances. Recent developments in gene sequencing may also make it possible to distinguish between main and secondary anticoagulant deficits in PVT [12].

Procoagulant and anticoagulant protein concentrations can be reduced in PVT patients for a variety of reasons, including hereditary or acquired thrombophilia, decreased hepatic blood flow, decreased production, hypertension of the portal vein, portosystemic shunts, clearing or utilization, portal pyemia, or additional local inflammatory diseases.

It is essential for diagnosis and the selection of the best course of action to accurately visualize the anomalies connected to PVT. Approximately a third of PVT instances include the portal vein becoming cavernous. The absence of high-level echoes in the porta hepatis region (also known as the "diamond sign"), the presence of multiple serpiginous venous channels surrounding the portal vein, and failure to visualize the extrahepatic portal vein are three ultrasonographically detectable signs of PVT [13].

Diagnostic CT with dynamic contrast enhancement is the most efficient way to identify PVT and assess probable underlying illnesses. Vessel wall rim augmentation and the presence of a filling deformity that partially or totally restricts the vessel lumen are two typical CT findings [14].

PVT might present with modest signs and symptoms that are frequently related to the underlying condition. An ancient thrombosis may be indicated in some circumstances by the presence of a well-developed cavernoma, although a prior PVT may be linked to a newly superimposed thrombus, which can result in acute symptoms. Doppler ultrasonography may not be as effective in detecting venous collateral proliferation and cavernoma as abdominal MRI [15]. Malignancy screening is crucial in PVT situations as it might complicate matters. Common follow-up consequences include bleeding, portal hypertensive gastrointestinal disease, and esophageal and stomach varices. Particularly in individuals without cancer or cirrhosis, portal hypertensive gastropathy is common [16]. Therefore, endoscopic screening should be performed on all PVT patients. Addressing the underlying causes, avoiding thrombosis extension, and regaining portal vein patency are the main objectives of treatment for both acute and chronic PVT. The main method for reopening the portal vein at the moment is anticoagulant treatment. However, there is no established standard; hence, its applicability is still up for discussion. Early therapy beginning is linked to a better outcome in patients with acute PVT. Although thrombolytic therapy has a lesser effectiveness and a higher risk of death than conservative treatment, it may nevertheless be useful [17]. Generally, surgical thrombectomy is not advised. For liver transplant recipients with acute PVT or when anticoagulant therapy is inadequate, other methods including a transjugular intrahepatic portosystemic shunt should be considered [4]. The prognosis for PVT in patients who are not cirrhotic and who do not have cancer is favorable, with an average survival rate of 92% after one year and 76% after five years [3,16].

## Conclusions

In conclusion, our case is exceptional as it involved a young male who had protein C deficiency, which is unusual in PVT situations. Advanced imaging methods are becoming more and more important for the identification and treatment of PVT, with better outcomes associated with early anticoagulant medication intervention. Rarely is surgical thrombectomy advised. Non-cirrhotic and non-neoplastic PVT patients have encouraging survival rates despite comorbidities and risk factors, highlighting the importance of prompt diagnosis and treatment.
